# Dexmedetomidine Attenuates Neurotoxicity in Developing Rats Induced by Sevoflurane through Upregulating BDNF-TrkB-CREB and Downregulating ProBDNF-P75NRT-RhoA Signaling Pathway

**DOI:** 10.1155/2020/5458061

**Published:** 2020-06-20

**Authors:** Yunxia Dong, Wei Hong, Zhiyin Tang, Yan Gao, Xiuying Wu, Hongtao Liu

**Affiliations:** ^1^Department of Anesthesiology, Shengjing Hospital of China Medical University, Shenyang, China; ^2^Department of Ultrasound, The Third Affiliated Hospital of Liaoning University of Traditional Chinese Medicine, Shenyang, China; ^3^Department of Anesthesiology, The First Affiliated Hospital of Hebei North University, Zhangjiakou, China

## Abstract

To investigate the mechanism dexmedetomidine in relieving the neurotoxicity of a developing brain induced by sevoflurane. Sprague-Dawley rats, 6 days old, were randomly divided into three groups. Rats in the control group were inhaled with air after injection of normal saline; rats in the sevoflurane group were injected with normal saline and inhaled with 3% sevoflurane for 2 h in three consecutive day; rats in the dexmedetomidine group were inhaled with 3% sevoflurane after intraperitoneal injection of dexmedetomidine 25 *μ*g/kg. WB results showed that mBDNF, pTrkB/TrkB, and CREB were significantly decreased in the hippocampus of the sevoflurane group, which are significantly upregulated in the dexmedetomidine group. In the sevoflurane group, proBDNF, P75NRT, and RhoA were significantly increased, which were significantly lower than those in the dexmedetomidine group than those in the sevoflurane group. The expression BDNF was downregulated in the sevoflurane group, while the proBDNF was upregulated in the sevoflurane group. In the Morris water maze test, the escape latency of the sevoflurane group was significantly prolonged. In sevoflurane groups, the number of crossing platform was significantly reduced, the synaptic protein decreased significantly, and this effect was reversed in rats of the dexmedetomidine group. Dexmedetomidine could reduce synaptic plasticity decline in developing rats induced by sevoflurane, through downregulating the proBDNF-p75NTR-RhoA pathway and upregulating BDNF-TrkB-CREB.

## 1. Introduction

The human brain has a rapid development period from the last three months of the embryo to the first three years after birth. If molecular structure changes, it will have an effect on the learning and memory ability [1, 2]. There are many reports stating that long-term, repeated, or high-dose sevoflurane anesthesia in neonatal rats can cause changes in nervous system function and affect the long-term learning and memory and cognitive function [3–5]. The FDA warned that repeated or prolonged (more than 3 h) use of anesthetics and sedatives may impair the brain of fetus and children less than 3 years [6].

Dexmedetomidine is a novel, highly selective, highly specific *α*2 adrenergic receptor agonist which is widely used as a sedative drug in clinic. Recent studies have shown that dexmedetomidine has neuroprotective effects such as on nerves, heart muscles, and kidneys [7–10]. Some studies have demonstrated that dexmedetomidine can attenuate the expression of brain-derived neurotrophic factor (BDNF) in the brain caused by isoflurane, thereby reducing the impact of synaptic plasticity [11–13]. This article explores the mechanism by which dexmedetomidine can alleviate the developmental neurological damage caused by sevoflurane, reduce learning and memory impairment, which paves the foundation for clinical use.

## 2. Materials and Methods

### 2.1. Material

#### 2.1.1. Animals

A total of 30 Sprague-Dawley (SD) rats (License number SCXK (Liao) 2015-0001), including 10 males and 20 females, weighing 220-250 g, were housed in a light-dark cycle at room temperature (24 ± 1°C) for 14:10 cycles under free access to water and food. When the females are pregnant, they are kept in separate cages until they give birth naturally. The day of birth of the child was recorded as day 0 (P0). Male rats in each litter were randomly selected for this study in the sixth day (P6).

### 2.2. Establishment of Sevoflurane Injury Model and Pretreatment with Dexmedetomidine

The animal protocol was approved by the Animal Welfare Ethics Committee of Shengjing Hospital of China Medical University (No. 2019PS010K). Male rats at P6 were randomly selected from each litter. Rats were injected peritoneally with dexmedetomidine 25 *μ*g/kg or the same amount of saline 20 minutes before exposure to sevoflurane or air control. During the experiment, a group of 12 rats were placed in a transparent glass anesthesia box (30 cm∗20 cm∗20 cm) for 2 hours per day. An internal heating lamp was used to keep the temperature inside the box at 37°C. After rats were naturally awakened, the mother rats were placed in cages. On P7 and P8, rats were treated as the same as on P6.

### 2.3. Western Blot Analysis

The hippocampus tissue was added to the lysate as 1 mg/10 *μ*L, and the hippocampus tissue was cut and sheared. The tissue was thoroughly pulverized, left on ice for 30 min, and centrifuged at 12000 g for 20 min at 4°C, and then, the supernatant was collected. The BCA protein concentration measures were used. Then, proteins were diluted to 40 *μ*g/10 *μ*L and loaded to 10 *μ*L on 10% sodium dodecyl sulfate-polyacrylamide gel electrophoresis (SDS-PAGE). After being separated on SDS-PAGE, proteins were transferred to the PVDF membrane. The PVDF membrane was placed in 5% skim milk for 4 h at room temperature, and the membrane was washed 3 times. The primary antibodies were added: anti-BDNF (1 : 2000, Abcam, Cambridge, UK), anti-proBDNF (1 : 500, Alomone Labs, Israel), P75NRT (1 : 2000, Cell Signaling Technology, USA), TrkB (1 : 1000, ABclonal, Woburn, MA, USA), p-TrkB (1 : 1000, ABclonal, Woburn, MA, USA), CREB (1 : 1000, Cell Signaling Technology, USA), anti-RhoA (1 : 500, Proteintech, China), PSD95 (1 : 500, Proteintech Biotechnology, Chicago, IL, USA), SYP (1 : 1000, Abcam, USA), anti-*β*-actin (1 : 500, Proteintech, China), and GAPDH (1 : 1000, Cell Signaling Technology, Boston, MA, USA). The membrane was kept overnight at a 4°C shaker, and the membrane was washed 3 times with TBST 10 min each and was added a second antibody (1 : 5000, Zhongshan Jinqiao Co., Ltd., China). The membrane was stored at room temperature for 90 min and washed 3 times for 10 min each. The membrane was added with the chemical luminescent substrate after 5 min, exposed in a C300 illuminator. ImageJ analysis software was used to analyze the results.

### 2.4. Immunofluorescence Assay

The prepared brain slices were washed with 50 mM PBS for 3 times for 10 min each, and then, the slices were incubated in 3% H_2_O_2_ for 30 min to block endogenous peroxidase activity, followed by immersion in blocking solution for 2 h and then incubated overnight with BDNF antibody (1 : 200, Abeam, Cambridge, UK) and proBDNF (1 : 50, Alomone Labs, Israel). To verify the expression of BDNF and proBDNF, we used rat anti-neuronal nuclear antibodies (1 : 200; Millipore, Billerica, MA, USA) for counterstaining on the same slice. Slices were then incubated with FITC-conjugated goat anti-rabbit secondary antibody (Vector Laboratories Inc., Burlingame, CA, USA) and FITC-conjugated goat anti-mouse secondary antibody (Jackson Immuno Research Laboratories Inc., West) for 2 h. The coverslips were fixed using a fluorescent mounting medium and were captured using a confocal laser scanning microscope of LSM 510 META used to take Fluorescence images.

### 2.5. Morris Water Maze (MWM) Experiment

Three groups were housed until P28; 10 rats in each group were examined by MWM experiments. The MWM equipment is a circular swimming pool. The escape latency experiment was performed in the first five days, four quadrants per day for five consecutive days, once in each quadrant. The device automatically recorded the time of the rats finding the hidden platform, and after 15 s being kept on the platform, rats were removed from the platform. If the platform was not found in 90 s, the rat was guided to the platform and stayed for 15 seconds. After five-day training, the platform was taken out and the rats were allowed to swim freely for 90 s to record the number of crossing through the platform. All videos, images, and data are recorded and processed using an automatic tracking system (Noldus, Holland).

### 2.6. Statistical Analysis

Data are expressed as the mean ± SEM, and multiple comparisons were calculated using one-way ANOVA followed by the Tukey test. GraphPad Prism 6 software (version 6.0; GraphPad Software, Inc.) and SPSS 22.0 software were used to analyze all data. *P* < 0.05 was used to consider the difference statistically significantly.

## 3. Results

### 3.1. Dexmedetomidine Alleviated the Decline in Learning and Memory Ability during Puberty after Sevoflurane Exposure in Developing Rats

**Figure 1:**
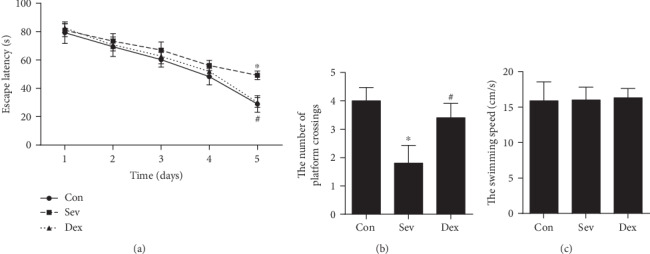
Dexmedetomidine alleviated the decline in learning and memory ability during puberty after sevoflurane exposure in developing rats. (a) The escape latency. (b) The number of crossing platforms on the sixth day. (c) Swimming speed. ^∗^Compared with the control group, *P* < 0.05. ^#^Compared with the Sev group, *P* < 0.05.

On the fifth day, the escape latency of the sevoflurane group was significantly prolonged (Figure 1(a), *P* < 0.05). The number of crossing platforms on the sixth day was significantly reduced (Figure 1(b), *P* < 0.05). There was no significant difference in swimming speed among the three groups (Figure 1(c), *P* < 0.05). Multiple exposure to sevoflurane during brain development in rats can affect learning and memory in adolescent rats, while dexmedetomidine can reverse the adverse effects of sevoflurane on learning and memory in the brain. We concluded that the pretreatment of dexmedetomidine can alleviate the long-term learning and memory impairment caused by sevoflurane.

### 3.2. Dexmedetomidine Improves Hippocampal Synaptic Protein Levels during Puberty after Sevoflurane Exposure in Developing Rats

**Figure 2:**
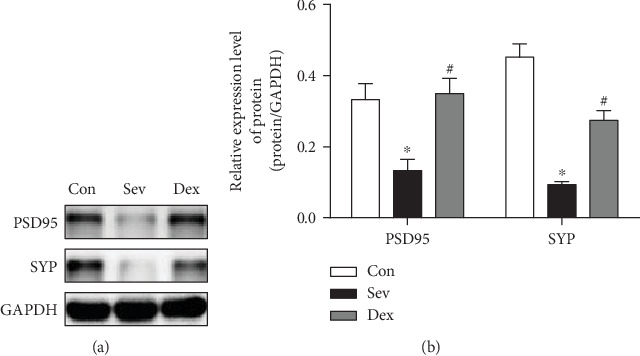
Dexmedetomidine improves hippocampal synaptic protein levels of sevoflurane exposure in developing rats. (a) Western blot band. (b) Bar graph of Western blot. ^∗^Compared with the control group, *P* < 0.05. ^#^Compared with the Sev group, *P* < 0.05.

Although the number of neurons in the brain cannot increase after birth, the density of neurons and the synaptic circuits formed by neurons has been continuously modified [14]. The synaptic density is important for the brain's ability in learning and remember. The proteins representing synaptic density mainly include postsynaptic density protein 95 (PSD95) and synaptophysin (SYP). Our study found that the hippocampal synaptic protein was still lower when the sevoflurane-inhaled rats reached puberty (32 days of birth) (Figure 2). This result was consistent with our previous research and other literature reports [15–18]. We found that preinjection of dexmedetomidine before sevoflurane exposure can alleviate the decline of long-term hippocampal synaptic protein SYP and PSD95 after sevoflurane anesthesia, suggesting that the protection of dexmedetomidine can last for long term.

### 3.3. The Pretreatment of Dexmedetomidine Could Increase the Level of Hippocampal Mature Brain-Derived Neurotrophic Factor mBDNF in Rats Exposed by Sevoflurane Increase

**Figure 3:**
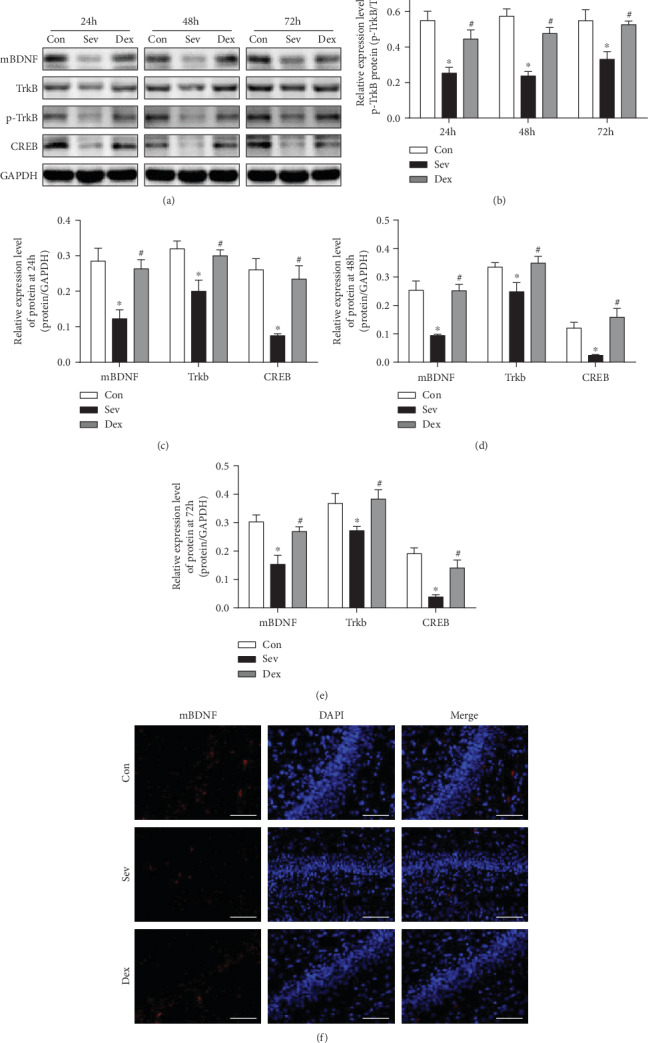
Dexmedetomidine could increase the level of mBDNF and relieve the inhibition of the BDNF-TrkB-CREB pathway caused by sevoflurane in the hippocampus. (a) Western blot band. (b–e) Bar graph of Western blot. (f) Immunofluorescence of mBDNF (scale bar = 50 *μ*m). ^∗^Compared with the control group, *P* < 0.05; ^#^Compared with the Sev group, *P* < 0.05.

The lack of BDNF can significantly reduce synaptic plasticity, which in turn affects synaptic development and growth, leading to decreased learning and memory. Our study found that BDNF, TrkB, and CREB levels in the hippocampus were significantly decreased after sevoflurane exposed in developing rats (*P* < 0.05). Pretreatment with dexmedetomidine significantly ameliorated the decreasing of hippocampal mBDNF, p-TrkB, TrkB, and CREB protein (Figures 3(a)–3(e)). Immunofluorescence results showed that mBDNF expression was lowest in the hippocampal CA1 region in the sevoflurane group, while mBDNF expression in the dexmedetomidine group was significantly higher than that in the sevoflurane group (Figure 3(f)). It indicated that dexmedetomidine increased the expression of mBDNF in the brain, thus activating the mBDNF-TrkB-CREB pathway and ameliorating the abnormal expression of mBDNF caused by sevoflurane.

### 3.4. Dexmedetomidine Inhibits proBDNF-P75NRT-RhoA Signaling Pathway Caused by Sevoflurane in the Hippocampus

**Figure 4:**
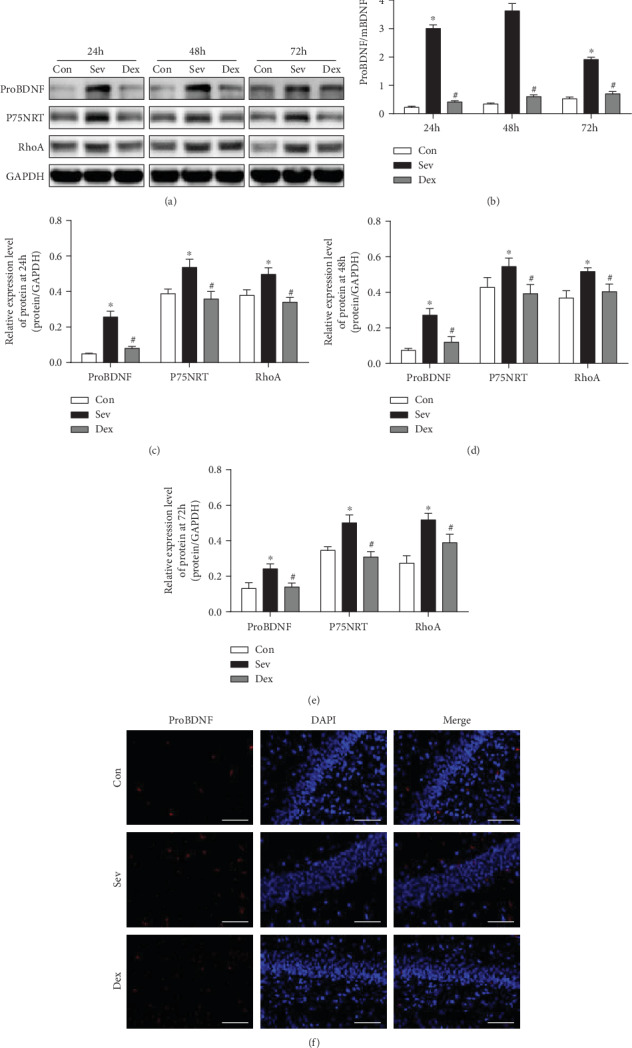
Dexmedetomidine could decrease the level of proBDNF and restore the ratio of proBDNF/mBDNF and alleviates activation of the proBDNF-P75NRT-RHOA pathway after sevoflurane. (a) Western blot band. (b) ProBDNF/mBDNF. (c–e) Bar graph of Western blot. (f) Immunofluorescence of proBDNF (scale bar = 50 *μ*m). ^∗^Compare with the control group, *P* < 0.05; ^#^Compared with the Sev group, *P* < 0.05.

The pretreatment of dexmedetomidine could relieve the obstacles of proBDNF cleavage in the hippocampus caused by sevoflurane exposure, which could reduce the ratio of proBDNF/mBDNF, and inhibit the proBDNF-P75NRT-RhoA signaling pathway. The BDNF precursor, proBDNF, is proteolytically cleaved into a mature form of BDNF. Many reports have confirmed proBDNF has intrinsic biological function, which is completely opposite to mBDNF [19]. ProBDNF can preferentially bind to p75NTR, which in turn activates RhoA, promotes apoptosis, and collapses of nerve cone, thus affecting synaptic plasticity [20–22]. We have shown that the proBDNF increased in the hippocampus after sevoflurane exposure, especially the ratio of proBDNF/mBDNF which is significantly increased. But dexmedetomidine can reduce the expression of proBDNF in the hippocampus and decrease the ratio of proBDNF/mBDNF. At the same time, P75NRT and RHOA increased significantly after sevoflurane exposure, and the expression of P75NRT and RHOA became normal after pretreatment of dexmedetomidine (Figure 4).

## 4. Discussion

A recent high-quality clinical randomized controlled trial reported that patients in an intensive care unit, pretreated with low-dose dexmedetomidine, could significantly reduce the incidence of delirium within 7 d after surgery and reduce postoperative patients' anxiety [23]. Basic experimental studies have found that dexmedetomidine can prevent learning and memory impairment caused by different brain injury models [24–26]. In recent years, some teams have confirmed that dexmedetomidine can counteract neuropathy or brain function changes in puberty caused by sevoflurane, a commonly used anesthetic drug in children, by reducing oxidative stress and reducing apoptosis [27, 28]. In our study, dexmedetomidine has a neuroprotective effect on the brain after sevoflurane exposure, and this protection can continue until puberty, relieving the reduction of learning and memory ability in puberty caused by sevoflurane. Dexmedetomidine provided neuroprotection against sevoflurane-induced neuroapoptosis in a dose-dependent manner. However, several reports describe how dexmedetomidine lacks neurotoxicity even at extremely high doses such as 75 *μ*g/kg. Pretreatment once with dexmedetomidine (75 *μ*g/kg) provides a similar effect to that of applying 25 *μ*g/kg [29, 30]. However, it has been reported recently that large doses of dexmedetomidine combined with sevoflurane may cause respiratory depression, carbon dioxide retention, asphyxia, death, and other adverse reactions, which may be due to different conditions of model preparation [28]. Therefore, we chose 25 *μ*g/kg for intraperitoneal administration at 20 min before sevoflurane exposure for three consecutive days. There were no death of rats and adverse effects of respiratory depression during the experiment. On the 20th day after the experiment, the protection of learning and memory ability can be observed and synaptic proteins were significantly attenuated in the hippocampal region of developing rats. Thus, 25 *μ*g/kg of dexmedetomidine was sufficient to induce neuroprotection.

As many studies have shown that the main reason for the changes in learning and memory ability caused by inhaled anesthetics is mainly due to the prominent plasticity of brain neurons such as synaptic density or reticular formation rather than neuronal abnormalities caused by apoptosis or oxidative stress [31, 32]. BDNF plays an important role in the regulation of synaptic plasticity, which is the basis of the brain's ability in learning and remembering. Thus, the main cause of inhaled anesthetics leading to brain learning and memory is the decline of BDNF in the brain [33, 34]. Our previous study found that dexmedetomidine reversed the abnormal expression of NR2A, EAAT1, and NR2B caused by sevoflurane and increased MLP2 expression, which was through the BMP/SMAD signaling pathway [25, 35]. We believe that dexmedetomidine protection on the brain during development after sevoflurane exposure is not only through the antiapoptosis and antioxidative stress. In this study, we found that the level of mBDNF in the hippocampus decreased after multiple exposure with sevoflurane and the level of BDNF-TrkB-CREB-related proteins was downregulated, which is the key pathway that determines synaptic plasticity [36, 37]. We clearly found that the levels TrkB and CREB proteins decreased. This recent decline in protein after modeling could cause the reduction of PSD95 and SYP levels in long term. PSD95 and SYP, markers of presynaptic and postsynaptic components, are involved in synapse formation and reconstruction [38–42]. Decreased protein expression means the loss of synapses, hippocampal synaptic plasticity decreases after multiple anesthesia with sevoflurane, and this effect can continue to puberty, accompanied by decreased synaptic protein levels during adolescence and decline of learning and memory function. After the treatment of dexmedetomidine, the decrease of mBDNF, TrkB, and CREB protein after sevoflurane exposure was alleviated, synaptic proteins were showed to return to normal, and thus, the learning and memory ability was improved at 28 d after modeling. These results suggested that dexmedetomidine may restore abnormalities in long-term synaptic protein expression and behavioral abnormalities by improving the BDNF-TrkB protective pathway.

At the same time, we found that the proBDNF in the hippocampus increased after sevoflurane exposure; especially, the ratio of proBDNF/mBDNF increased significantly. This result is consistent with the head team's report that isoflurane could increase the proBDNF in vitro [43]. The cleavage of proBDNF is reduced leading to mBDNF decreasing; thus, proBDNF is relatively more abundant than normal condition. Previous literatures have shown that there is a reverse regulation of increased proBDNF in different pathological models. ProBDNF binds to P75NRT and activates the downstream RhoA-Rock pathway, causing cascades of neurite outgrowth and filopodial growth cones by activating RhoA [44–47]. The recently pathway protein changes could affect the long-term synaptic protein changes and behavioral changes. Our observations suggest that proBDNF collapses neurite outgrowth and filopodial growth cones by activating RhoA through the p75NTR signaling pathway. After pretreatment of dexmedetomidine, we found that the ratio of proBDNF/mBDNF was significantly decreased, the pathway proteins were significantly decreased, and this effect continued into puberty. Therefore, dexmedetomidine can alleviate the increase of proBDNF after sevoflurane exposure and decrease RhoA protein. It may be the protective effect of the developmental brain.

There is a limit in this experiment. We did not use pathway agonists and inhibitors or knockout rats to observe the specific targeting of dexmedetomidine but only detected the protein expression changes to speculate the mechanism of action of dexmedetomidine.

These data demonstrated that pretreatment with dexmedetomidine may offer a strategy for the protection of the neonatal brain during sevoflurane anesthesia.

We first reported that the brain protection of dexmedetomidine may be associated with restoring the ratio of proBDNF/mBDNF after sevoflurane exposure. Then, the hippocampal synaptic proteins were corrected during puberty, and memory impairment was repaired after sevoflurane exposure. Dexmedetomidine could alleviate sevoflurane-induced proBDNF aggregation and thus increase mature BDNF and activate the BDNF-directed TrkB-CREB pathway. Dexmedetomidine also reduces proBDNF levels, thus alleviates sevoflurane activation of the proBDNF-P75NRT-RHOA pathway, thereby reduces the effects of sevoflurane exposure on synaptic plasticity, and reduces the decline in learning and memory caused by sevoflurane.

## Data Availability

The datasets used and/or analyzed during the current study are available from the corresponding author on reasonable request.
